# *SynchLink*: an iOS app for ISPyB

**DOI:** 10.1107/S1600576714017531

**Published:** 2014-09-04

**Authors:** Helen Mary Ginn, Ghita Kouadri Mostefaoui, Karl Erik Levik, Jonathan Mark Grimes, Martin Austin Walsh, Alun William Ashton, David Ian Stuart

**Affiliations:** a Diamond House, Diamond Light Source, Harwell Science and Innovation Campus, Didcot, Oxfordshire OX11 0DE, UK; b Division of Structural Biology, The Wellcome Trust Centre for Human Genetics, University of Oxford, Roosevelt Drive, Oxford, Oxfordshire OX3 7BN, UK

**Keywords:** remote data collection, synchrotron radiation, macromolecular crystallography, laboratory information management systems (LIMS)

## Abstract

The macromolecular crystallography (MX) user experience at synchrotron radiation facilities continues to evolve, with the impact of developments in X-ray detectors, computer hardware and automation methods making it possible for complete data sets to be collected on timescales of tens of seconds. Data can be reduced in a couple of minutes and in favourable cases structures solved and refined shortly after. The information-rich database ISPyB, automatically populated by data acquisition software, data processing and structure solution pipelines at the Diamond Light Source beamlines, allows users to automatically track MX experiments in real time. In order to improve the synchrotron users’ experience, efficient access to the data contained in ISPyB is now provided *via* an iOS 6.0+ app for iPhones and iPads. This provides users, both local and remote, with a succinct summary of data collection, visualization of diffraction images and crystals, and key metrics for data quality in real time.

## Abstract 2

An iOS app has been developed as a front end to ISPyB, a laboratory information system for macromolecular crystallography synchrotron beamlines.

## 1. Introduction   

Synchrotron radiation has revolutionized the field of macromolecular crystallography (MX) (Dauter *et al.*, 2010 ▶), so that today the vast majority of structure determinations use synchrotron radiation. As a result of advances in detector developments, improved X-ray optics, sample automation and associated software, measurement of diffraction data is now possible in time frames of tens of seconds. There is now also a widespread availability of automated sample mounting (a summary of robotics for MX is given on the RoboSync web site: http://smb.slac.stanford.edu/facilities/hardware/SAM/robosync) and automated pipelines for data integration and scaling (Holton & Alber, 2004 ▶; Minor *et al.*, 2006 ▶; Monaco *et al.*, 2013 ▶; Vonrhein *et al.*, 2011 ▶; Winter, 2010 ▶). The speed at which experimental data are collected and the rapidity of subsequent online data analysis make experiment tracking *via* databases almost obligatory. However, ensuring efficient use of synchrotron beamtime while dealing with the logistics of user access to the facilities has always been challenging, and developing robust protocols for remotely accessing beamlines has provided many advantages to both the facilities and users (Soltis *et al.*, 2008 ▶). To increase the efficiency of beamtime usage while simplifying the logistics of access, many facilities encourage users to form larger collaborative groups which can be geographically disperse, allowing large numbers of samples to be assessed in allocated blocks of time. This idea of categorizing users into block allocation groups (BAGS) originated at the European Synchrotron Radiation Facility (ESRF) and has proved very successful. Key to the success of this access model has been ISPyB (Delageniere *et al.*, 2011 ▶; Beteva *et al.*, 2006 ▶), a laboratory information system that is in place at the ESRF and Diamond Light Source. ISPyB enables users to manage and track, through a web-based interface, transport of their samples to the synchrotron as well as providing reports of data collections and analyses as they are executed in real time at the beamline.

Extending the reach of ISPyB to smartphones and tablets provides users with the ability to access their synchrotron data from any location on an easily portable device and further enhances the user experience. Therefore, we have developed an iOS 6.0+ app, *SynchLink*, to allow MX users of Diamond to interact with ISPyB to monitor their experiments in real time. We chose iOS in the first instance to mitigate the number of variables, limiting the types of hardware to support the iPhone/iPad (iOS 6.0+), and therefore we have used the native Apple development tools, *i.e.* the Objective-C language and the Xcode integrated development environment (https://developer.apple.com/xcode/).

## 2. Description   

### 2.1. Design of graphical user interface   

**Figure 1:**
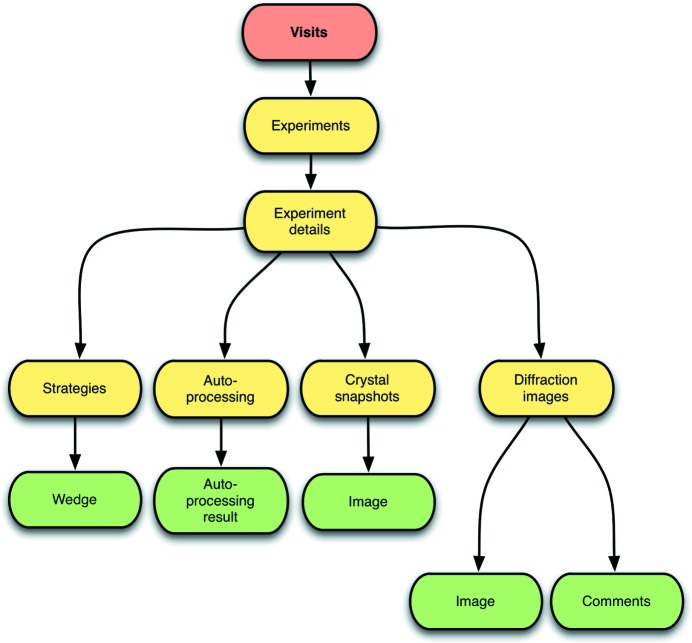
Summary of the individual screens within the navigation system of *SynchLink*. Starting from the top level where a user can see all synchrotron visits and their associated proposal, users can drill down through an experiment to see data collection strategy information, autoprocessing results, crystal snapshots or diffraction patterns.

**Figure 2:**
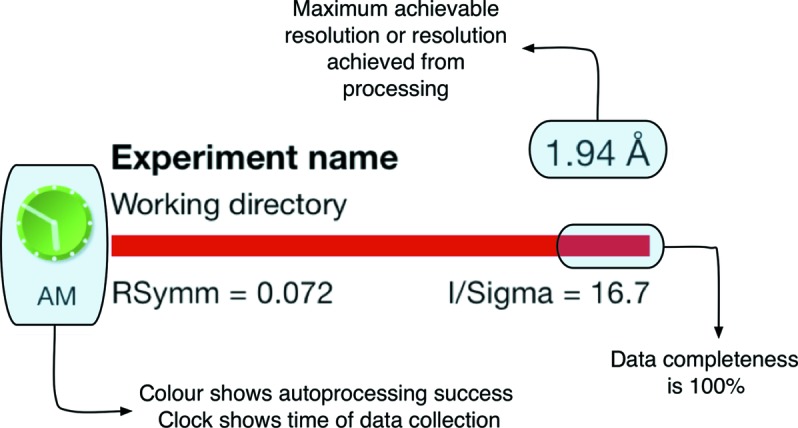
Detail of the information content provided by *SynchLink* through the iPhone screen. The key parameters associated with the data collection and reduction statistics, such as file names, directory paths, data quality parameters *R*_symm_ and 〈*I*/σ(*I*)〉, and data completeness, are presented in a single-screen list for each synchrotron visit to provide fast, direct and informative summaries to the user.

**Figure 3:**
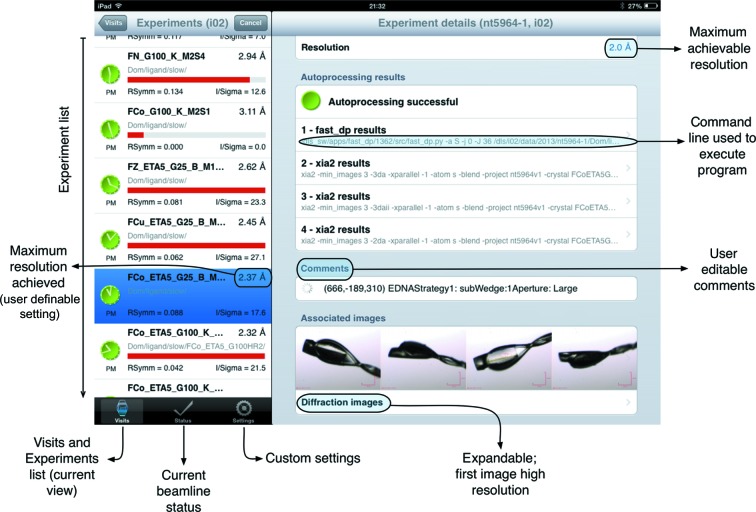
Screenshot from the iPad *SynchLink* screen (for a successful data collection), illustrating how the app exploits the larger screen area of the device. The left panel iPhone view is augmented by the right panel view, which provides both numerical and image data of the selected data collection highlighted in blue. In the above example four data processing runs which were executed automatically are displayed, as well as four snapshots of the mounted sample taken at 90° intervals. Images (*e.g.* crystal images) can be touched to save and to enlarge, whilst the autoprocessing results are also action buttons that bring up a summary of the results. Other touch screen points and features are annotated.

To provide ease of use with a small handheld device such as an iPhone, *SynchLink* is arranged as a content drill-down scheme, starting from the highest level screen which lists each visit to the synchrotron, leading to a list of data collections for that visit, and finally down to the associated data and data analysis (Fig. 1 ▶). We have developed separate interfaces to use the differing real estate on the phone and tablet devices optimally. In the case of the iPhone a single view is visible and a simple drill-down navigation system allows clutter-free information to be viewed. The crucial information from each data collection has been carefully designed to be presented on the top level page of each synchrotron visit. This allows a complete summary of each data collection and associated data analysis for a synchrotron trip to be reviewed on a single screen from just one screen touch (Fig. 2 ▶). This is achieved by use of graphics and colours. For instance, the time at which an experiment was performed has been compressed into an analogue clock face, whose colour reflects the success of the data collection strategy or autoprocessing programs (grey for unknown, red for failed, yellow for indexing success and green for full autoprocessing success). The completeness of the data is represented by the length of the red bar across the entry for the experiment. Finally *R*_symm_, 〈*I*/σ(*I*)〉 and the resolution (user configured to be either that achieved by the autoprocessing or the resolution at the edge of the detector) are shown to provide a quick assessment of data quality (Fig. 2 ▶). From this top level summary the user can then easily drill down to view extended details of the experiments, autoprocessing or data collection strategy results, and diffraction and other images associated with the data collection experiments (Fig. 1 ▶). In the case of the iPad the larger screen allows a dual page view, which simultaneously displays both the data collection list and a more detailed view of data from a selected data collection (Fig. 3 ▶).

A key consideration in the design of the graphical user interface was ease of location of a specific data collection or series of experiments through keyword input. Hence, search functionality has been enabled both from the top level (all synchrotron trips) and from within a specific trip or visit. Searching matches data collections or experiments with a particular name or working directory, or any of the associated comments. In conjunction with a well structured directory and file naming system, this provides an extremely rapid and powerful facility for searching results for particular projects with a bare minimum of information. Furthermore, since comments for data collections are editable from within the app, the use of defined tags can enhance the search facility, enabling searches for particular experiments or experimental outcomes at a later date.

### 2.2. Information presented   

The *SynchLink* app has detailed pages presenting the relevant data from data collections and data reduction (Fig. 1 ▶). On drilling down to an individual data collection, experimental parameters are shown (including rotation per image, number of images, % transmission, beamsize), and *SynchLink* provides further drill-down content including crystal snapshots, diffraction patterns, indexing and autoprocessing results, and suggested data collection strategies on the lowest level. Crystal snapshots and diffraction patterns can be seen as thumbnails or clicked on to provide higher-resolution images. One diffraction image per data collection is available at the highest resolution. Multi-touch gestures (zooming and panning) make it easier to see the potentially tiny reflections produced on low-divergence beamlines with pixel array detectors. For data collections of less than 20 images, which are categorized as data collection strategy experiments, the most likely point group and cell dimensions are presented and a range of data collection strategies are calculated using *EDNA* (Incardona *et al.*, 2009 ▶). Each of these strategies can be expanded to see the individual rotation wedges suggested. For full data collections (20 or more images), autoprocessing results (if successful) provide data quality measurements using *FastDP* and *xia2* (Winter, 2010 ▶): completeness, 〈*I*/σ(*I*)〉 and *R*_symm_, both overall and in the highest resolution bin, the maximum resolution integrated, the estimated space group, and unit-cell dimensions.

*SynchLink* also provides a service to show the current status of the storage ring and each beamline, prioritizing MX beamlines at the top of the list.

## 3. Discussion   

Two important issues we have addressed when developing *SynchLink* are the speed of access and minimizing usage of mobile data networks.

Owing to the nature of internet access on smartphones and tablets, often using mobile data networks with data limits, care has been taken to ensure that *SynchLink* downloads the minimum possible data in XML format to avoid compromising responsiveness and to minimize loading times. Furthermore, *SynchLink* has been designed with ‘one step ahead of the user’ in mind. Data for the next screen in the drill-down hierarchy is loaded while the user is on the previous screen, as long as the content size is sufficiently low to keep traffic to a minimum on mobile data networks. This is aided by the ability of the web service to execute database queries that join information from multiple tables, thereby reducing the total number of calls the iOS device must make to retrieve information.

To avoid draining the battery charge on a small mobile device, it is inadvisable to keep an open connection to a server. *SynchLink* therefore uses multiple single connections that are rapidly opened and closed to save battery life. However, this means that every connection must be authenticated. Full authentication is time consuming as it involves communication with an authentication server, so once completed, the client then obtains a time-limited token which can be used for subsequent authentication. After one hour of inactivity, full authentication is once again required.

## 4. Conclusions   

We hope that *SynchLink* will enable users with iOS devices to access their data more simply and efficiently than by using the ISPyB web interface, and will be particularly valuable when a computer and/or wired internet connection is unavailable. Access to real-time results from the beamline *via* a personal mobile device or tablet should improve productivity for researchers following live data collection remotely. *SynchLink* has been made available to users on the iOS App Store and can be freely downloaded in any of the countries where the Apple App Store is available.
